# Functional and structural analysis of non-synonymous single nucleotide polymorphisms (nsSNPs) in the MYB oncoproteins associated with human cancer

**DOI:** 10.1038/s41598-021-03624-x

**Published:** 2021-12-17

**Authors:** Shu Wen Lim, Kennet JunKai Tan, Osman Mohd Azuraidi, Maran Sathiya, Ee Chen Lim, Kok Song Lai, Wai-Sum Yap, Nik Abd Rahman Nik Mohd Afizan

**Affiliations:** 1 Department of Cell and Molecular Biology, Faculty of Biotechnology and Biomolecular Sciences, 43400 Serdang, Selangor Malaysia; 2 Faculty of Applied Sciences, UCSI University, No. 1, Jalan Menara Gading UCSI Height, 56000 Cheras, Kuala Lumpur, Malaysia; 3 School of Pharmacy, Monash University Malaysia, Jalan Lagoon Selatan, 47500 Bandar Sunway, Selangor Malaysia; 4 Health Sciences Division, Abu Dhabi Women’s College, Higher Colleges of Technology, 41012 Abu Dhabi, United Arab Emirates

**Keywords:** Bioinformatics, Cancer genomics, Cancer, Computational biology and bioinformatics, Genetics, Pathogenesis

## Abstract

MYB proteins are highly conserved DNA-binding domains (DBD) and mutations in MYB oncoproteins have been reported to cause aberrant and augmented cancer progression. Identification of MYB molecular biomarkers predictive of cancer progression can be used for improving cancer management. To address this, a biomarker discovery pipeline was employed in investigating deleterious non-synonymous single nucleotide polymorphisms (nsSNPs) in predicting damaging and potential alterations on the properties of proteins. The nsSNP of the MYB family; *MYB*, *MYBL1*, and *MYBL2* was extracted from the NCBI database. Five in silico tools (PROVEAN, SIFT, PolyPhen-2, SNPs&GO and PhD-SNP) were utilized to investigate the outcomes of nsSNPs. A total of 45 nsSNPs were predicted as high-risk and damaging, and were subjected to PMut and I-Mutant 2.0 for protein stability analysis. This resulted in 32 nsSNPs with decreased stability with a DDG score lower than − 0.5, indicating damaging effect. G111S, N183S, G122S, and S178C located within the helix-turn-helix (HTH) domain were predicted to be conserved, further posttranslational modifications and 3-D protein analysis indicated these nsSNPs to shift DNA-binding specificity of the protein thus altering the protein function. Findings from this study would help in the field of pharmacogenomic and cancer therapy towards better intervention and management of cancer.

## Introduction

MYB oncoproteins; *MYB*, *MYBL1*, and *MYBL2* plays important roles in the modulation of cell cycle, and dysregulation in these genes have been implicated with abberant behaviours of the tumour cells. The key functions of MYB proteins are mainly in cell growth and differentiation, thereof mutations within these genes are predicted to be a potential source for oncogenesis^1^. Numerous studies have reported mutation of MYB proteins toward pathogenesis of human cancers, especially acute lymphoblastic leukaemia (ALL)^2^, paediatric low-grade gliomas^3^, cancers of the gastrointestinal tract^4–6^ and breast cancer^7^.Growing evidences of MYB oncoproteins and cancers necessitates an in-depth understanding at molecular level in unravelling its pathogenesis towards cancer.

The use of computational predictors to identify damaging non-synonymous single nucleotide polymorphisms (nsSNPs) towards understanding disease-causing role offers a time- and cost-effective alternative^8^. nsSNPs are changes in an amino acid that could disrupt the structure and stability of protein thus potentially increasing susceptibility towards certain disease^9,10^. Evaluating the influence of nsSNPs on the protein is important in determining its effect towards characterisation of the disease^11^. Recent studies employing computational approaches have also revealed the effectiveness of nsSNPs in understanding the molecular mechanisms of numerous diseases^12–14^.

Considering the pathological role of MYB oncoproteins towards cancer, functional and structural analysis of MYB oncoproteins still remains vague. Therefore, this study sets to examine the role of nsSNPs of *MYB*, *MYBL1*, and *MYBL2* genes through bioinformatics tools in understanding its pathogenesis toward cancer. This approach enables the differentiation of pathogenic mutations from an abundance of variations, narrowing down to highly significant variant for further investigation using laboratory validation^15^. In this study, MYB oncoproteins were subjected to multi-level functional and structural analysis in determining their pathogenicity. In brief, this involves (1) nsSNP analysis, where sequence evolutionary conservation information and structure-based information to determine damaging nsSNPs, (2) Prediction of protein stability, which evaluates the energetics of the folded and unfolded state of proteins in determining if the protein is stable, and (3) prediction of post-translational modifications. Each of these tools employs a different machine-learning algorithm to predict the outcomes.

## Results

### Retrieval of nsSNPs in *MYB* family genes

NCBI dbSNP was used to retrieve the SNPs of *MYB* oncoproteins. A total of 51,862 SNPs were extracted from the NCBI database of which; 13,632 of *MYB*, 17,770 of *MYBL1*, and 20,460 of *MYBL2*. Out of these SNPs, 1503 were nsSNPs.

### Identification of deleterious nsSNPs in *MYB* family genes

The nsSNPs were then subjected to five different tools (PROVEAN, SIFT, Polyphen2, SNPs&GO and PhD-SNP) that has different prediction algorithms to identify nsSNP with significant deleterious effects which could affect the biological structure and the function of MYB proteins. Forty-eight nsSNPs were identified as “pathogenic” or “damaging” by all tools, hence classified at “high-risk” (Table 1).

**Table 1:** High risk nsSNPs identified in *MYB* family genes by i*n silico* tools.

SNP ID	AA change	PROVEAN		SIFT		PolyPhen-2		SNPs&GO		PhD-SNP	
		Pred^a^	Sc	Pred^b^	Sc	Effect^c^	Sc	Pred^d^	RI	Pred^e^	RI
**MYB**											
rs1302072057	R73L	Del	− 3.80	Dmg	0	Pro.dmg	1	Disease	4	Disease	7
rs1302072057	R73Q	Del	− 6.65	Dmg	0	Pro.dmg	1	Disease	5	Disease	8
rs866246271	C78Y	Del	− 10.45	Dmg	0	Pro.dmg	0.999	Disease	7	Disease	9
rs1246761830	K84E	Del	− 3.80	Dmg	0.001	Pro.dmg	0.998	Disease	5	Disease	4
rs1231582413	P94R	Del	− 8.55	Dmg	0.001	Pos.dmg	0.608	Disease	3	Disease	1
rs1776412940	W95L	Del	− 12.35	Dmg	0	Pro.dmg	1	Disease	4	Disease	7
rs1361650612	G111S	Del	− 5.69	Dmg	0	Pro.dmg	1	Disease	5	Disease	7
rs1316378738	A158E	Del	− 4.74	Dmg	0	Pos.dmg	0.577	Disease	7	Disease	6
rs1583273308	R176Q	Del	− 3.80	Dmg	0	Pro.dmg	1	Disease	6	Disease	8
rs1179275735	N183S	Del	− 4.58	Dmg	0	Pro.dmg	1	Disease	7	Disease	5
rs1335964521	R191Q	Del	− 3.75	Dmg	0	Pro.dmg	1	Disease	2	Disease	5
rs1444007668	K192E	Del	− 3.75	Dmg	0	Pro.dmg	0.999	Disease	7	Disease	6
rs1777011832	D286Y	Del	− 6.18	Dmg	0	Pro.dmg	1	Disease	4	Disease	7
rs1247338239	W406C	Del	− 5.30	Dmg	0.001	Pro.dmg	0.985	Disease	0	Disease	5
rs1247579811	P574S	Del	− 6.02	Dmg	0	Pro.dmg	1	Disease	0	Disease	5
rs775717051	A594P	Del	− 3.37	Dmg	0.001	Pro.dmg	1	Disease	1	Disease	6
rs756830286	G603S	Del	− 4.53	Dmg	0.007	Pro.dmg	1	Disease	0	Disease	5
**MYBL1**											
rs1367098628	R68C	Del	− 7.60	Dmg	0	Pro.dmg	1	Disease	3	Disease	7
rs767355502	R68H	Del	− 4.75	Dmg	0	Pro.dmg	0.996	Disease	1	Disease	6
rs1472109411	P83T	Del	− 7.60	Dmg	0	Pos.dmg	0.811	Disease	3	Disease	3
rs766676175	G122S	Del	− 5.69	Dmg	0.002	Pro.dmg	0.975	Disease	3	Disease	5
rs768073245	R156W	Del	− 6.55	Dmg	0.002	Pro.dmg	1	Disease	2	Disease	5
rs1299187617	R160H	Del	− 4.74	Dmg	0	Pro.dmg	0.990	Disease	2	Disease	5
rs866260709	S175Y	Del	− 3.62	Dmg	0	Pro.dmg	1	Disease	0	Disease	2
rs1281804000	R185Q	Del	− 3.30	Dmg	0.003	Pro.dmg	0.978	Disease	0	Disease	3
rs1809689475	E265K	Del	− 3.34	Dmg	0.005	Pro.dmg	0.957	Disease	2	Disease	6
rs1225867137	M276T	Del	− 4.79	Dmg	0.006	Pro.dmg	0.992	Disease	2	Disease	6
rs1808814297	P512R	Del	− 6.44	Dmg	0	Pro.dmg	1	Disease	1	Disease	6
rs1361362325	C514W	Del	− 5.98	Dmg	0.002	Pro.dmg	1	Disease	1	Disease	6
rs770108773	A562E	Del	− 3.54	Dmg	0.001	Pro.dmg	1	Disease	0	Disease	6
rs777095803	A565P	Del	− 2.90	Dmg	0.003	Pro.dmg	1	Disease	1	Disease	7
rs777150665	G596R	Del	− 3.07	Dmg	0.001	Pro.dmg	1	Disease	0	Disease	5
rs1281394929	G721E	Del	− 5.73	Dmg	0.007	Pro.dmg	1	Disease	5	Disease	6
**MYBL2**											
rs748449655	L57R	Del	− 4.37	Dmg	0.001	Pro.dmg	0.999	Disease	8	Disease	2
rs1228232756	R64C	Del	− 6.64	Dmg	0	Pro.dmg	1	Disease	8	Disease	5
rs867195152	G102D	Del	− 6.10	Dmg	0	Pro.dmg	0.995	Disease	8	Disease	6
rs1164247754	R116Q	Del	− 3.35	Dmg	0.022	Pro.dmg	1	Disease	8	Disease	6
rs1323182096	R124C	Del	− 7.06	Dmg	0	Pro.dmg	1	Disease	8	Disease	7
rs1300383239	N127D	Del	− 4.52	Dmg	0.006	Pro.dmg	0.971	Disease	2	Disease	4
rs1295676923	G166V	Del	− 8.00	Dmg	0	Pos.dmg	0.946	Disease	8	Disease	7
rs968286439	R167K	Del	− 2.63	Dmg	0	Pro.dmg	0.999	Disease	7	Disease	5
rs1271670254	D169G	Del	− 5.87	Dmg	0	Pro.dmg	1	Disease	7	Disease	7
rs1438994955	S178C	Del	− 4.01	Dmg	0	Pro.dmg	1	Disease	3	Disease	3
rs781229138	G188C	Del	− 6.21	Dmg	0.003	Pro.dmg	0.966	Disease	6	Disease	5
rs1171631148	E552V	Del	− 5.52	Dmg	0.001	Pro.dmg	1	Disease	7	Disease	2
rs776972688	G530R	Del	− 5.97	Dmg	0.001	Pro.dmg	1	Disease	7	Disease	2
rs779332836	G669R	Del	− 6.13	Dmg	0	Pro.dmg	1	Disease	6	Disease	3
rs776117094	R682W	Del	− 5.78	Dmg	0	Pro.dmg	1	Disease	5	Disease	1

*AA* amino acid, *Pred* prediction, *TI* tolerance index, *Sc* score, *Del* deleterious, *Dmg* damaging, *Pro.dmg* probably damaging, *Pos.dmg* possibly damaging, *RI* reliability index.

^a^PROVEAN: Del (Sc < − 2.5).

^b^SIFT: Dmg (Sc ≤ 0.05).

^c^PolyPhen-2: Pos.dmg (0.453 ≤ Sc ≤ 0.956), Pro.dmg (0.957 ≤ Sc ≤ 1.0).

^d^SNPs&GO: Disease (Probability > 0.5).

^e^PhD-SNP: Disease (Probability > 0.5).

### Verification of high risk nsSNPs by PMut

The selected damaging nsSNPS were then submitted to PMut server to determine the probability score and the status of prediction of the resultant protein due to mutations. Table 2 shows the prediction scores and statuses. All *MYBL2* nsSNPs were predicted as high-risk, whereas 16 nsSNPs and 14 nsSNPs from *MYB* and *MYBL1* genes were also identified as “disease”. The “disease” status indicates that the mutated proteins are predicted to be pathogenic.

**Table 2:** Predictions of high risk nsSNPs in MYB oncoproteins by PMut, I-Mutant 2.0, and ConSurf.

Protein	nsSNP ID	Mutation	PMut		I-Mutant 2.0			ConSurf	
			Score and percentage	Prediction^a^	Stability	RI	DDG (kcal/mol)	Conservation score^b^	Prediction
MYB	rs1302072057	R73L	0.86 (91%)	Disease	Decrease	9	− 0.90	9	Highly conserved and exposed (f)
	rs1302072057	R73Q	0.73 (87%)	Disease	Decrease	9	− 1.38	9	Highly conserved and exposed (f)
	rs866246271	C78Y	0.79 (89%)	Disease	Decrease	2	− 0.07	9	Highly conserved and buried (s)
	rs1246761830	K84E	0.52 (79%)	Disease	Decrease	1	− 0.30	9	Highly conserved and exposed (f)
	rs1231582413	P94R	0.72 (86%)	Disease	Decrease	7	− 0.42	9	Highly conserved and exposed (f)
	rs1776412940	W95L	0.86 (91%)	Disease	Decrease	7	− 0.99	9	Highly conserved and exposed (f)
	rs1361650612	G111S	0.80 (89%)	Disease	Decrease	9	− 1.03	9	Highly conserved and exposed (f)
	rs1316378738	A158E	0.82 (90%)	Disease	Decrease	7	− 1.28	9	Highly conserved and buried (s)
	rs1583273308	R176Q	0.86 (91%)	Disease	Decrease	8	− 0.51	9	Highly conserved and exposed (f)
	rs1179275735	N183S	0.82 (90%)	Disease	Decrease	4	− 0.04	9	Highly conserved and exposed (f)
	rs1335964521	R191Q	0.77 (88%)	Disease	Decrease	9	− 1.13	9	Highly conserved and exposed (f)
	rs1444007668	K192E	0.55 (80%)	Disease	Decrease	5	− 0.95	9	Highly conserved and exposed (f)
	rs1777011832	D286Y	0.64 (84%)	Disease	Increase	2	0.11	2	Exposed
	rs1247338239	W406C	0.65 (84%)	Disease	Decrease	6	− 1.49	7	Buried
	rs1247579811	P574S	0.63 (84%)	Disease	Decrease	9	− 1.01	9	Highly conserved and exposed (f)
	rs775717051	A594P	0.65 (84%)	Disease	Increase	2	− 1.01	9	Highly conserved and buried (s)
	rs756830286	G603S	0.50 (82%)	Neutral	Decrease	6	− 1.52	9	Highly conserved and exposed (f)
MYBL1	rs767355502	R68C	0.39 (86%)	Neutral	Decrease	3	− 0.73	9	Highly conserved and exposed (f)
	rs767355502	R68H	0.86 (91%)	Disease	Decrease	7	− 1.31	9	Highly conserved and exposed (f)
	rs1472109411	P83T	0.81 (89%)	Disease	Decrease	4	0.08	9	Highly conserved and exposed (f)
	rs766676175	G122S	0.76 (88%)	Disease	Decrease	7	− 0.52	9	Highly conserved and exposed (f)
	rs768073245	R156W	0.82 (90%)	Disease	Decrease	7	− 1.04	9	Highly conserved and exposed (f)
	rs1299187617	R160H	0.77 (88%)	Disease	Decrease	9	− 1.17	9	Highly conserved and exposed (f)
	rs866260709	S175Y	0.76 (88%)	Disease	Increase	2	− 0.64	9	Highly conserved and buried (s)
	rs1281804000	R185Q	0.67 (85%)	Disease	Decrease	9	− 1.44	4	Highly conserved and exposed (f)
	rs1809689475	E265K	0.63 (83%)	Disease	Decrease	5	− 0.38	6	Exposed
	rs1225867137	M276T	0.32 (89%)	Neutral	Decrease	5	− 0.62	9	Exposed
	rs1808814297	P512R	0.86 (91%)	Disease	Decrease	9	− 1.25	6	Highly conserved and exposed (f)
	rs1361362325	C514W	0.63 (84%)	Disease	Decrease	5	− 1.06	9	Buried
	rs770108773	A562E	0.78 (88%)	Disease	Decrease	5	− 0.76	9	Highly conserved and buried (s)
	rs777095803	A565P	0.53 (80%)	Disease	Increase	0	− 1.85	8	Highly conserved and exposed (f)
	rs777150665	G596R	0.53 (80%)	Disease	Decrease	7	− 1.87	9	Highly conserved and exposed (f)
	rs1281394929	G721E	0.82 (90%)	Disease	Increase	2	− 0.27	9	Highly conserved and exposed (f)
MYBL2	rs748449655	L57R	0.66 (85%)	Disease	Decrease	6	− 0.76	9	Highly conserved and buried (s)
	rs1228232756	R64C	0.78 (88%)	Disease	Decrease	4	− 0.66	9	Highly conserved and exposed (f)
	rs867195152	G102D	0.86 (91%)	Disease	Decrease	5	− 1.45	9	Highly conserved and exposed (f
	rs1164247754	R116Q	0.88 (92%)	Disease	Decrease	7	0.13	9	Highly conserved and exposed (f)
	rs1323182096	R124C	0.89 (92%)	Disease	Decrease	6	0.41	9	Highly conserved and exposed (f)
	rs1300383239	N127D	0.85 (91%)	Disease	Decrease	6	− 0.73	9	Highly conserved and exposed (f)
	rs1295676923	G166V	0.88 (92%)	Disease	Increase	0	− 0.89	9	Highly conserved and exposed (f)
	rs968286439	R167K	0.87 (91%)	Disease	Decrease	9	− 0.98	9	Highly conserved and exposed (f)
	rs1271670254	D169G	0.79 (89%)	Disease	Decrease	6	− 1.04	9	Highly conserved and exposed (f)
	rs1438994955	S178C	0.53 (80%)	Disease	Decrease	2	− 1.33	9	Highly conserved and exposed (f)
	rs781229138	G188C	0.80 (89%)	Disease	Decrease	8	− 2.28	5	Exposed
	rs1171631148	G530R	0.76 (88%)	Disease	Decrease	4	− 1.69	9	Highly conserved and exposed (f)
	rs776972688	E552V	0.71 (86%)	Disease	Increase	2	0.08	9	Highly conserved and exposed (f)
	rs779332836	G669R	0.82 (90%)	Disease	Decrease	7	− 1.24	9	Highly conserved and exposed (f)
	rs776117094	R682W	0.82 (90%)	Disease	Decrease	5	0.65	9	Highly conserved and exposed (f)

*RI* reliability index, *DDG* free energy change value, *(f)* predicted functional residue (highly conserved and exposed), *(s)* predicted structural residue (highly conserved and buried).

^a^PMut: Disease (Score > 0.5), Neutral (Score ≤ 0.5).

^b^ConSurf: highly variable (1), highly conserved (9).

### Determination of protein structural stability by I-Mutant 2.0

The structural stability of resultant proteins was predicted by I-Mutant 2.0. The output of I-Mutant 2.0 was expressed in free energy change value (DDG) and reliability index (RI). In total, 41 nsSNPs were confirmed to cause decrease in stability to the resultant proteins, however only 32 nsSNPs were predicted to have a DDG value < − 0.5, indicating its greater impact towards the proteins.

### Evolutionary conservation analysis by ConSurf

The evolutionary conservation was determined through subjecting the mutated protein sequences to the ConSurf web server. A total of thirty-six nsSNPs of *MYB* family genes were identified as functional, highly conserved with exposed amino acid residues, whereas six nsSNPs were predicted as structural, highly conserved and buried. On the contrary, a total of four nsSNPs were predicted to be exposed but not functional, while two nsSNPs were predicted as buried and not functional (Table 2).

The R73L, R73Q, K84E, P94R, W95L, G111S, R176Q, N183S, R191Q, K192E, P574S, R68H, P83T, G122S, R156W, R160H, R185Q, P512R, G596R, R64C, G102D, R116Q, R124C, N127D, R167K, D169G, S178C, G530R, G669R, and R682W mutations were predicted to be pathogenic, highly conserved and exposed with decreased protein stability, indicating the most significant damaging effect. Hence, these 30 high risk nsSNPs were proceeded with post-translational modification sites prediction.

### Prediction of post-translational modification (PTM) sites

**Figure 1:**
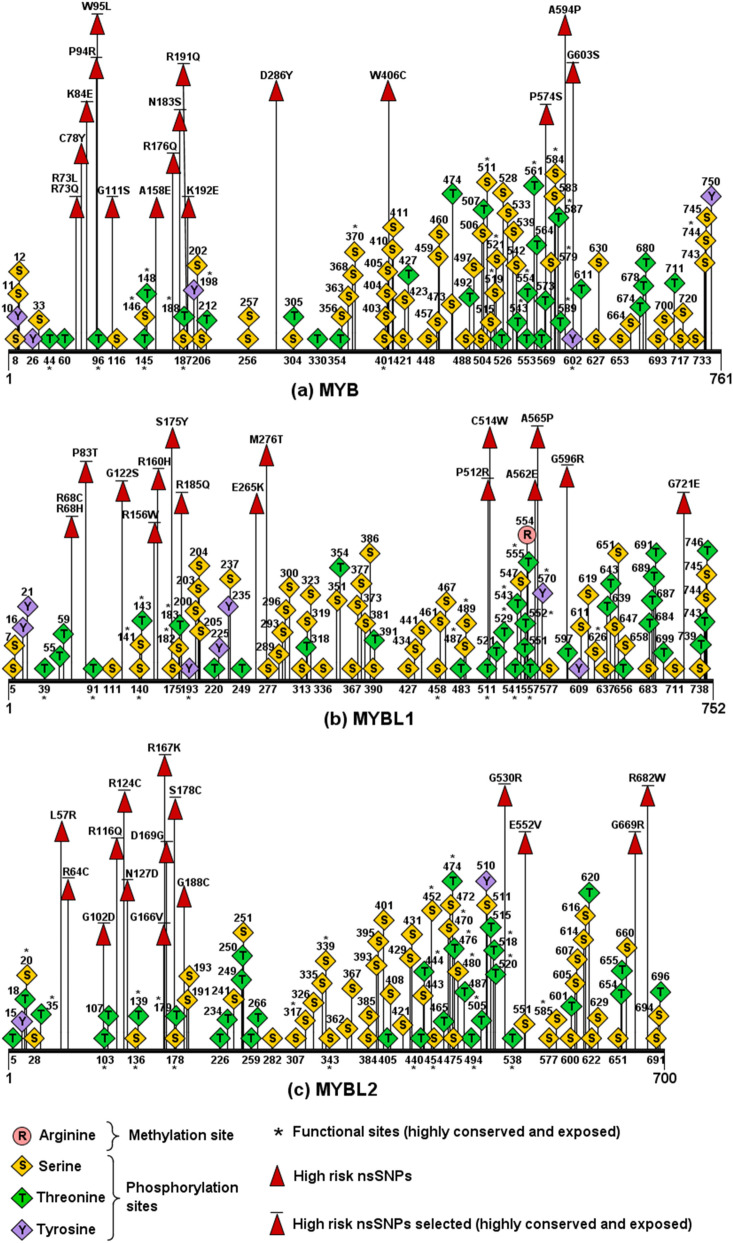
Putative PTM sites and high risk nsSNPs in MYB family proteins. (**a**) MYB had 90 common phosphorylation sites (Ser:56, Thr:29, Tyr:5), of which 22 were functional sites. (**b**) MYBL1 had 1 common methylation site and 90 common phosphorylation sites (Ser:50, Thr:33, Tyr:7), of which 21 were functional sites. (**c**) MYBL2 had 81 common phosphorylation sites (Ser:48, Thr:31, Tyr:2), of which 24 were functional si.

Post-translational modification (PTM) refers to the process where proteins undergo chemical modification to become functional and participate in respective cellular activities^16^. Putative methylation sites in the MYB family proteins and the 30 high risk nsSNPs were predicted using MusiteDeep and GPS-MSP 1.0. Only the wild-type R554 in MYBL1 was predicted as the common site (Fig. 1). Phosphorylation sites in the native and 30 mutated proteins were predicted using NetPhos 3.1 and GPS 5.0. NetPhos 3.1 predicted 268 residues in the three proteins to have phosphorylation potentials. A total of 386 residues in the proteins were found to be phosphorylated using the GPS 5.0, and 261 phosphorylation sites in all proteins were identified using both the tools, which consisted of 154 serines, 93 threonines, and 14 tyrosines (Fig. 1). However, only two wild-type reisudes (S175 and S178) and four mutant residues (S111, S183, S574, and S122) were the common sites present in the high risk nsSNPs. The ubiquitylation predictors employed in this study were BDM-PUB and UbiNet 2.0. BDM-PUB found 110 ubiquitylation sites at lysine residues in the family proteins. Whereas, only the wild-type K84 and K109 were predicted by UbiNet 2.0 in MYB. Both BDM-PUB and UbiNet 2.0 did not have common findings in all three proteins. After assembling the results from ConSurf and various PTM tools, a total of five high risk nsSNPs; G111S, N183S, P574S, G122S, and S178C containing putative phosphorylation sites were selected to proceed with comparative 3D modelling (Table 3).

**Table 3:** High risk nsSNPs selected by considering ConSurf and PTM predictions.

Protein	SNP ID	Mutation	ConSurf		PTM
			Conservation score^a^	Prediction	
MYB	rs1361650612	G111S	9	e, f	Phosphorylation
	rs1179275735	N183S	9	e, f	Phosphorylation
	rs1247579811	P574S	9	e, f	Phosphorylation
MYBL1	rs766676175	G122S	9	e, f	Phosphorylation
MYBL2	rs1438994955	S178C	9	e, f	Phosphorylation

*b* buried residue, *s* predicted structural residue (highly conserved and buried), *e* exposed residue, *f* predicted functional residue (highly conserved and exposed).

^a^Highly variable (1), highly conserved (9).

### Comparative modelling of wild-type MYB family proteins and their mutant structures

**Figure 2:**
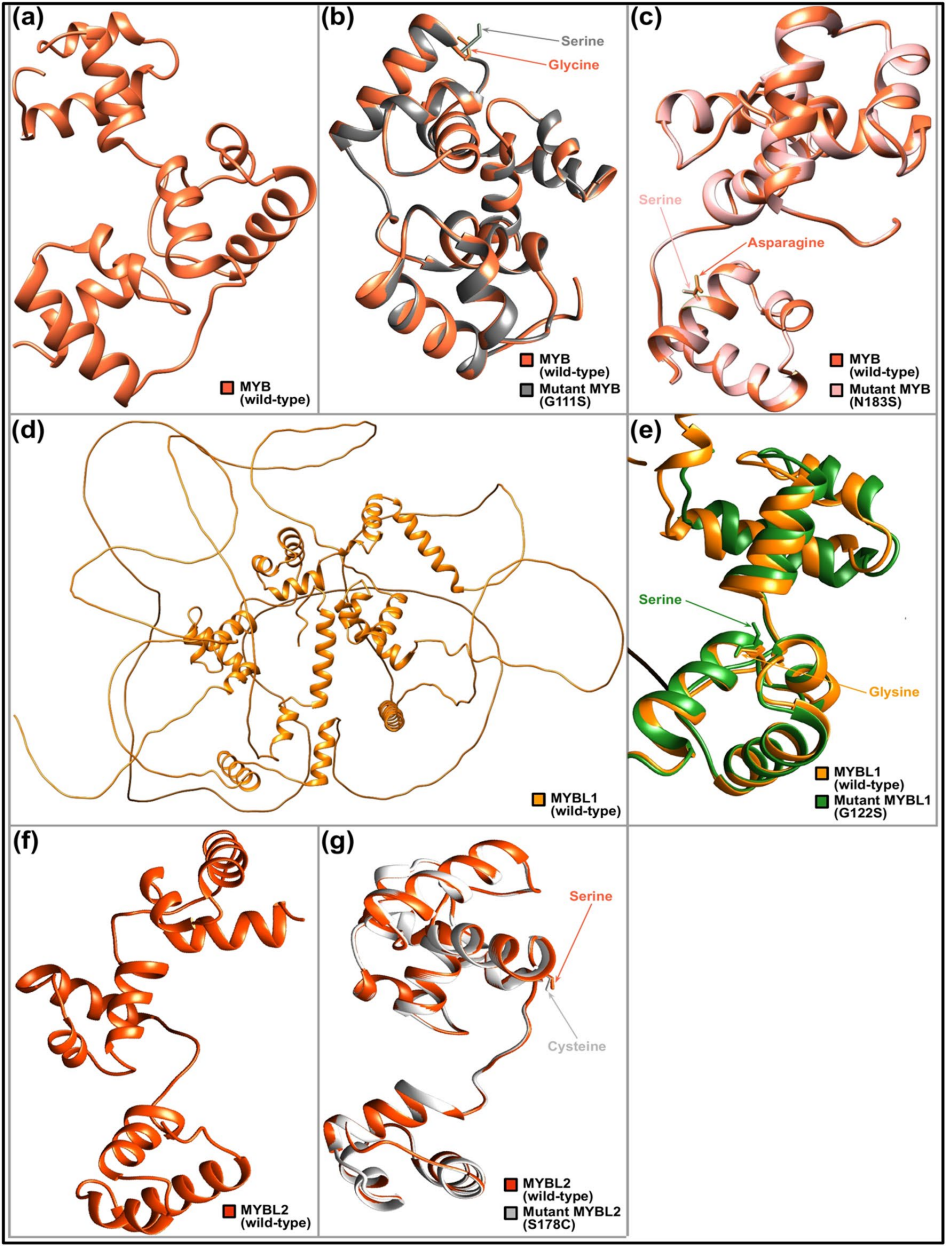
Structural comparison of wild-type MYB family proteins with their mutant forms. (**a**) 3D model of wild-type MYB protein. (**b**) Superimposed structures of wild-type MYB protein and its mutant having mutation from Glycine to Serine at position 111. (**c**) Superimposed structures of wild-type MYB protein and its mutant having mutation from Asparagine to Serine at position 183. (**d**) 3D model of wild-type MYBL1 protein. (**e**) Superimposed structures of wild-type MYBL1 protein and its mutant having mutation from Glycine to Serine at position 122. (f) 3D model of wild-type MYBL2 protein. (**g**) Superimposed structures of wild-type MYBL2 protein and its mutant having mutation from Serine to Cysteine at position 178.This figure was generated using UCSF Chimera 1.15 (https://www.cgl.ucsf.edu/chimera/download.html).

To investigate if the five high risk nsSNPs substantially alter the resultant proteins, predictive 3D modelling was performed along with the structural comparisons between wild-type and mutant models. The c1h88C and c1mseC templates were used to predict the wild-type MYB family proteins and their mutant models, excluding the P574S structure as this mutant residue was not covered in either template. TM-align revealed all mutant models had values of TM-score = 0 and RMSD = 1, showing no structural variations from their wild-type forms (Table 4). SWISS-MODEL was used to construct the 3D models for the wild-type proteins and their mutants. The best template used for the MYB family protein structures was 1h88.1.C as most 3D models can be generated based on this template. The generated mutant models were validated by ERRAT, and models with the highest possible GMQE scores, QMEAN Z-scores, and ERRAT values were selected for structural comparisons (Table 4). These models were visualised in Chimera 1.15 and the corresponding mutation positions induced by the nsSNPs were affirmed (Fig. 2). The structural integrity of generated wild-type and mutant protein structures were further validated with Ramachandran plot through the dihedral angles calculated.

**Table 4:** TM-score, RMSD value, GMQE score, QMEAN Z-score, ERRAT value, and PROCHECK Ramachandran plot analysis of the selected protein models.

Protein	Model	TM-align		SWISS-MODEL		ERRAT	PROCHECK Ramachandran plot analysis			
		TM-score^a^	RMSD	GMQE score	QMEAN Z-score^b^	ERRAT value (overall quality factor)^c^	Residues in most favoured regions^d^	Residues in additional allowed regions^d^	Residues in generously allowed regions^d^	Residues in disallowed regions^d^
MYB	+	Nil	Nil	Nil	Nil	97.222	120 (87.6%)	17 (12.4%)	0 (0.0%)	0 (0.0%)
	G111S	1	0	0.15	0.55	99.3056	122 (88.4%)	15 (10.9%)	0 (0.0%)	1 (0.7%)
	N183S	1	0	0.16	0.72	98.6111	122 (89.1%)	15 (10.9%)	0 (0.0%)	0 (0.0%)
MYBL1	+	Nil	Nil	Nil	Nil	97.9167	123 (89.1%)	15 (10.9%)	0 (0.0%)	0 (0.0%)
	G122S	1	0	0.15	− 0.02	98.6014	123 (89.1%)	15 (10.9%)	0 (0.0%)	0 (0.0%)
MYBL2	+	Nil	Nil	Nil	Nil	96.5278	119 (86.2%)	19 (13.8%)	0 (0.0%)	0 (0.0%)
	S178C	1	0	0.16	0.19	96.5278	118 (85.5%)	20 (14.5%)	0 (0.0%)	0 (0.0%)

*TM-score* template modelling-score, *RMSD* root-mean-square deviation, *GMQE* global model quality estimation, *QMEAN* qualitative model energy analysis, *“*+*”* Wildtype.

^a^Random structural similarity (0.0 < TM-score < 0.30), both structures are within the same fold (0.50 < TM-score < 1.00).

^b^Low quality model (QMEAN Z-score ≤ − 4.0).

^c^Reliable model (ERRAT value > 85%).

^d^Number of residues (percentage of residues).

Wild-type and mutant PDB inputs were subjected to PROCHECK for analysis. The wildtype MYB has 120 residues (87.6%) in the most favored region, 17 residues (12.4%) in the additional allowed region. The more damaging mutants, G111S has 122 residues (88.4%) in the most favored region, 15 residues (10.9%) in the additional allowed region and 1 residue (0.7%) in the disallowed region, followed by N183S possesses 122 residues (89.1%) in the most favored region, 15 residues (10.9%) in the additional allowed region. In MYBL1, both G122S mutant and wildtype possess the same amino acid residue patterns, 123 residues (89.1%) in the most favoured region and 15 residues (10.9%) in the additional allowed region, indicating no significant changes in the alteration in the structure. Wildtype MYBL2 has 119 residues (86.2%) in the most favoured region and 19 residues (13.8%) in the additional allowed region. Mutant S178C in MYBL2 has 118 residues (85.5%) in the most favoured region and 20 residues (14.5%) in the additional allowed region as shown as in Table 4.

## Discussion

This study has successfully identified high-risk pathogenic nsSNPs in the MYB oncoproteins towards understanding its association with human cancer using an in-silico approach. *MYB* oncoproteins play crucial roles in multiple signalling pathways for cellular activities. A study by Andersson and colleagues^17^ showed that overexpressed wild-type MYB genes are normally benign, however, overexpression accompanied by gene alteration, dysregulated gene rearrangement or the incorrect oncoprotein binding onto enhancer region could promote tumorigenesis^1^. Despite the mutations in MYB oncoproteins being reported frequently in numerous cancers, the precise mechanisms of tumour initiations and/or maintenance remains vague. Therefore, examining the outcomes of deleterious nsSNPs of the *MYB* oncoproteins could potentially pave ways into a better understanding thus revealing its deleterious effects. Therefore, the aim of this study is to develop a bioinformatics pipeline in determining the most damaging nsSNPs and their effects on the structure and function of *MYB*, *MYBL1*, and *MYBL2* proteins.

A total of 51,862 SNPs were extracted from the NCBI dbSNP for the *MYB* family genes, of which 1503 were nsSNPs. Structural analysis using (PROVEAN, PolyPhen-2, SIFT, SNPs&GO, and PhD-SNP) and functional analysis using PMut resulted in 45 “high-risk” pathogenic nsSNPs. Next, the stability of these nsSNPs were determined using I-Mutant 2.0, where 41 nsSNPs were identified with “decreased stability”. Protein stability is one of the key features to determine if a protein is biologically active and functional^18^. Proteins with decreased stability due to mutation might give rise to tumorigenesis as the fitness level for normal proteins dropped and conferred the fitness for tumorigenic proteins^19^.

Evolutionary conservation of MYB protein residues were calculated using ConSurf. The evolutionary conservation of an amino acid indicates its natural tendency for mutation to take place and highly conserved and exposed amino acids that undergo mutations can be expected to be most deleterious^20^. Thirty-six nsSNPs were identified to be highly conserved and exposed by ConSurf with the conservation score of 9. Next, these nsSNPs were subjected to PTMs analysis to determine its effect on regulating functions and structures of proteins. The G111S, N183S, P574S, G122S, and S178C showed to harbour putative phosphorylation sites. These nsSNPs coinciding with the putative phosphorylation sites may cause functional impairment and destabilisation of the corresponding proteins, thereby enhancing PTM impairment. PTM plays a pivotal role in modulating various protein functions and expressions, therefore mutations in PTM sites could lead towards malfunctions of the protein’ regulatory mechanisms, contributing to cellular dysfunctions such as transformation into cancer cells^21^. Several studies showed that mutated residues at phosphorylation sites had led to detrimental alterations in the expressions and functions of *MYB*^22,23^ and *MYBL2*^24–26^. Among the four nsSNPs, G111S mutation in *MYB* was reportedly associated with uterine leiomyosarcomas^27^. Analysis using methylation and ubiquitylation predictors were also performed on the selected high risk nsSNPs. Only the R554 consensus methylation site was identified in *MYBL1*. As the results of methylation and ubiquitylation sites prediction were not in agreement for *MYB* and *MYBL2*, it was considered that both PTM sites were not predicted in these two proteins.

Structural alterations on the resulting proteins were constructed using the Phyre2 homology modelling tool. Protein 3-dimensional analysis offers a detailed insight into the associated molecular changes^28^. Two templates (c1h88C and c1mseC) were utilised to construct the protein models. These templates were selected based on high sequence similarities and high GMQE value, providing a high coverage. The TM-scores and RMSD values obtained for the four mutants suggest that the nsSNPs might not have a significant structural consequence on the proteins for TM-align to detect. Protein structure homology-modelling tool SWISS-MODEL was conducted to remodel the four nsSNPs for structure and function prediction. The template (1h88.1.C) was used as it has high sequence identity and desired coverage range. GMQE scores of the models were estimated to be around 0.15–0.16, indicating that the models cover only 15–16% of the targeted sequence. All models have QMEAN Z-scores greater than − 4.0, indicating the high quality of the models. Finally, ERRAT evaluation gives overall quality factors that approximately hundred to all models, indicating high quality models were built.

The G111S and G122S were identified to be located in the helix-turn-helix (HTH) myb-type 2 domain and S178C and N183S in the HTH myb-type 3 domain. These domains are important for the binding of DNA sequences and gene expression. This could cause activation and overexpression of the gene through loss of the C-terminal negative regulatory domain (C-myb)^29^. Previous studies have also reported that these could lead towards loss of the 3′ UTR binding sites thus negatively regulating *MYB* mRNA stability and translation^30,31^.

Thus, the nsSNPs identified within these regions may have a complete shift in the DNA-binding specificity, resulting in a pathogenic protein synthesis^32^.

## Methods

### Retrieving nsSNPs

**Figure 3:**
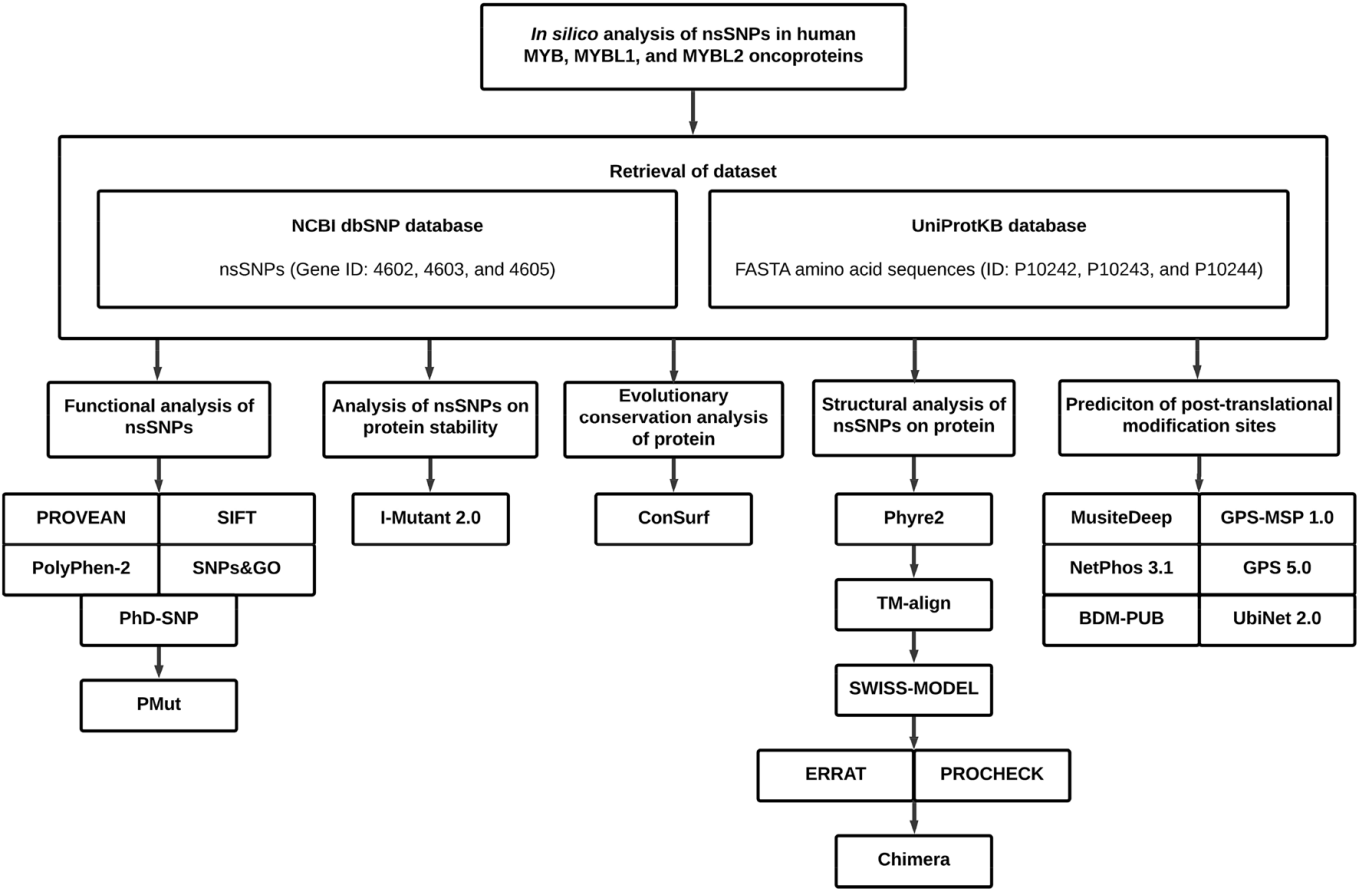
Diagrammatic representation of methodology.

nsSNPs of *MYB* (gene ID: 4602), *MYBL1* (gene ID: 4603), and *MYBL2* (gene ID: 4605) were extracted from NCBI (National Center for Biological Information) dbSNP database [https://www.ncbi.nlm.nih.gov/snp/]^33^. DNA sequences as well as other information related to the nsSNPs of each gene, including the SNP IDs, allele changes, positions, protein accession numbers, residue changes, and global minor allele frequencies (MAFs) were also retrieved from this database. A total of 490 *MYB*, 483 *MYBL1*, and 530 *MYBL2* nsSNPs were extracted respectively. The amino acid sequences of these genes (UniProtKB ID: P10242, P10243, and P10244) were obtained from the UniProtKB (Universal Protein Knowledgebase) database [https://www.uniprot.org/uniprot/] in FASTA format. Overview of the whole methodological approach is summarised in a schematic diagram (Fig. 3).

### Identifying deleterious nsSNPs

The functional effect of the nsSNPs were predicted through five bioinformatics tools; PROVEAN (Protein Variation Effect Analyser) embedded with SIFT (Sorting Intolerant From Tolerant) [http://provean.jcvi.org/genome_submit_2.php?species=human]^34–36^, PolyPhen-2 (Polymorphism Phenotyping v2) [http://genetics.bwh.harvard.edu/pph2/bgi.shtml]^37^, and SNPs&GO (Single Nucleotide Polymorphisms and Gene Ontology) embedded with PhD-SNP (Predictor of human Deleterious Single Nucleotide Polymorphisms) [https://snps.biofold.org/snps-and-go/snps-and-go.html]^38^. Those nsSNPs which were predicted to be deleterious by all five in silico tools were considered as “high-risk” nsSNPs and selected for further downstream analysis^39^. This ensured the stringency and accuracy of the results by incorporating the scores of all five computational tools to increase the precision of prediction.

### Validating the high risk nsSNPs

PMut [http://mmb.irbbarcelona.org/PMut/] was resorted to validate the pathological nature of the selected high risk nsSNPs^40^. This neural network-based tool includes 27,203 harmful and 38,078 benign mutations for 12,141 proteins. Prediction score ranging from 0 to 1 was computed along with the prediction percentage. The nsSNPs with a score of ≤ 0.5 are classified as neutral, whereas those with > 0.5 are predicted as disease-associated^40^.

### Determining protein stability

Protein stability of the nsSNPs were determined through I-Mutant 2.0 [https://folding.biofold.org/i-mutant/i-mutant2.0.html]^41^. This tool determines the increase decrease of stability change in mutated protein, and simultaneously estimates the corresponding values of free energy change (DDG). I-Mutant 2.0 uses a support vector machine method and a ProTherm-derived dataset, which is the most collective databank containing experimental thermodynamic data of free energy changes in mutated protein stability^41^. Along with these predictions, a reliability index (RI) ranging from 0 (lowest reliability) to 10 (highest reliability) was also computed by this web server.

### Protein evolutionary conservation analysis

ConSurf [https://consurf.tau.ac.il/] was used to predict the evolutionary conservation of each residue position in the native MYB proteins^41^. The prediction is based on an empirical Bayesian algorithm and the phylogenetic relations between close homologous sequences. For each amino acid position, a colorimetric conservation score between 1 and 9 is calculated by the tool and then classified as either a variable (1–4), intermediately conserved (5–6), or highly conserved residue (7–9). The exposed (on protein surface) or buried (inside protein core) status of each residue position in the protein structure is also determined. A functional residue is predicted when it is highly conserved and exposed, whereas a structural residue is predicted if it is highly conserved and buried^20,42^.

### Post‑translational modification sites prediction

The putative methylation sites at arginine and lysine residues in each MYB protein, were predicted using MusiteDeep [https://www.musite.net/]^43^ and GPS-MSP 1.0 (Group-based Prediction System-Methyl-group Specific Predictor Version 1.0) [http://msp.biocuckoo.org/online.php]^44^. Using a default cut-off of 0.5, the deep learning-based MusiteDeep predicts and labels the desired PTM sites in the sequence according to the confidence threshold^43^. As for GPS-MSP 1.0, types of mono, symmetrical di-, and asymmetrical di-methylation specific to arginines, as well as mono, di- and tri-methylation types specific for lysines were predicted ^45^. Phosphorylation sites in each MYB protein at serines, threonines, and tyrosines were predicted using NetPhos 3.1 [https://services.healthtech.dtu.dk/service.php?NetPhos-3.1]^46^. and GPS 5.0 (Group-based Prediction System Version 5.0) [http://gps.biocuckoo.cn/index.php]^47^. A higher score in GPS 5.0 indicates higher probability of residues getting phosphorylated. Then, BDM-PUB (Prediction of Ubiquitination Sites with Bayesian Discriminant Method) [http://bdmpub.biocuckoo.org/prediction.php]^48^ and UbiNet 2.0 [https://awi.cuhk.edu.cn/~ubinet/index.php]^49^ were employed to predict putative protein ubiquitination sites at the lysines in MYB family proteins. A balanced cut-off option and a threshold of 0.3 were selected for the BDM-PUB server to perform the prediction based upon Bayesian Discriminant Method (BDM)^50^.

### Examining the effects of nsSNPs with 3D protein modelling

The nsSNPs that were predicted as pathogenic, highly conserved with decreased protein stability, and possessing PTM sites were chosen to proceed with 3D protein modelling using 1h88.1.C template. To construct the 3D structures for wild-type and mutants MYB proteins, two distinct homology-modelling tools were employed: Phyre2 (Protein Homology/analogy Recognition Engine V 2.0) [http://www.sbg.bio.ic.ac.uk/phyre2/html/page.cgi?id=index]^51^ and SWISS-MODEL [https://swissmodel.expasy.org/]^52^. Each nsSNP was individually substituted into the respective sequence of each MYB protein and then submitted to Phyre2 for the creation of 3D mutant models based on selected templates. TM-align (Template Modelling-align) [https://zhanggroup.org/TM-align/]^53^ was utilised to investigate the similarities between the modelled wild-type and mutant protein structures by computing template modelling-score (TM-score) and root-mean-square deviation (RMSD) values. TM-score yields a result from 0 to 1, where 1 denotes a perfect match between both structures^54^. Precisely, 0.0 < TM-score < 0.30 indicates random structural similarity, whereas 0.50 < TM-score < 1.00 implies that both structures are within the same fold^55,56^. A lower TM-score and a higher RMSD value indicate a greater structural deviation of mutant models from those of wild-type^57^. To build the 3D models in SWISS-MODEL, templates were analysed and selected based on coverage, sequence identity, qualitative model energy analysis (QMEAN) Z-score, and global model quality estimation (GMQE) score. A QMEAN Z-score of ≤ − 4.0 denotes a low quality model^58^. The GMQE score, which ranges from 0 to 1, indicates the likely accuracy of the model constructed with that alignment and the target coverage^59^. Therefore, templates with higher sequence similarities and a higher GMQE value were prioritized, concurrent with the coverage of the mutation site in that template, thus, template 1h88.1.C was selected. The built models were then validated by ERRAT [https://saves.mbi.ucla.edu/] and PROCHECK Ramachandran plot analysis [https://saves.mbi.ucla.edu/] to estimate their structural quality. Then, the validated structures were viewed and superimposed using Chimera 1.15 [https://www.cgl.ucsf.edu/chimera/download.html].

## Conclusion

MYB family members are often aberrantly expressed in human cancers, suggesting that they could be important for tumour initiation and/or maintenance. In this study, a total of 30 nsSNPs were predicted as high-risk pathogenic, conserved with decreased stability, suggesting potential deleterious effect on the protein structure. Further PTM and 3D protein modeling indicated rs1361650612 (**G111S**), rs1179275735 (**N183S**), rs766676175 (**G122S**), and rs1438994955 (**S178C**) located within the helix-turn-helix (HTH) myb-type 2 and myb-type 3 domains were identified pathogenic with the ability to potentially cause great functional and stability impairment on the proteins. This study concise confidence that these findings could serve as a benchmark towards potential diagnostic and therapeutic interventions.

## Data Availability

The datasets generated during and/or analysed during the current study are available from the corresponding author on reasonable request.
